# Airborne bacterial species in indoor air and association with physical factors

**DOI:** 10.14324/111.444/ucloe.000056

**Published:** 2023-03-31

**Authors:** Anne Mette Madsen, Saloomeh Moslehi-Jenabian, Mika Frankel, John Kerr White, Margit W. Frederiksen

**Affiliations:** 1 National Research Centre for the Working Environment, Lersø Parkallé 105, 2100 Copenhagen Ø, Denmark; 2 Division of Clinical Microbiology, Karolinska University Hospital, Stockholm, Sweden

**Keywords:** *Bacillus megaterium*, bacteria, exposure, home environment, indoor air, indoor humidity, MALDI-TOF MS, *Paracoccus yeei*, room-to-room variation, seasonality

## Abstract

The aim of this study is to obtain knowledge about which cultivable bacterial species are present in indoor air in homes, and whether the concentration and diversity of airborne bacteria are associated with different factors. Measurements have been performed for one whole year inside different rooms in five homes and once in 52 homes. Within homes, a room-to-room variation for concentrations of airborne bacteria was found, but an overlap in bacterial species was found across rooms. Eleven species were found very commonly and included: *Acinetobacter lowffii*, *Bacillus megaterium, B. pumilus*, *Kocuria carniphila*, *K. palustris*, *K. rhizophila, Micrococcus flavus*, *M. luteus, Moraxella osloensis* and *Paracoccus yeei*. The concentrations of Gram-negative bacteria in general and the species *P. yeei* were significantly associated with the season with the highest concentrations in spring. The concentrations of *P. yeei*, *K. rhizophila* and *B. pumilus* were associated positively with relative humidity (RH), and concentrations of *K. rhizophila* were associated negatively with temperature and air change rate (ACR). *Micrococcus flavus* concentrations were associated negatively with ACR. Overall, this study identified species which are commonly present in indoor air in homes, and that the concentrations of some species were associated with the factors: season, ACR and RH.

## Introduction

Airborne bacteria in indoor environments are confirmed or presumed causal agents of various infectious diseases [1,2]. In addition, airborne bacteria are inflammogenic [3] and seem to be involved in either an increase or decrease in the risk of developing asthma and atopy [4,5]. Indoor work activities such as, for example, bed making [6,7] can aerosolise bacteria, but whether concentrations of airborne bacteria in a home are related to room type is not clear. Concentrations of airborne bacteria are often higher in homes than in offices [8], and since the Covid-19 pandemic, more office work is occurring from homes. Generally, studies comparing the indoor and outdoor levels of bacteria have found that the indoor : outdoor ratios are above 1 [9–14].

Exposure assessment using personal samplers, for example, the Gesamtstaubprobenahme (GSP) (CIS by BGI, Inc., Waltham, MA, USA), actively sampling airborne inhalable dust, is expected to be a good measure of personal exposure [15]. However, the sampling activity may interfere with everyday life in a home, due to, for example, noise of the sampling pump, and the necessity of the presence of a technician; thus only a few studies of this kind have been performed. Instead, bacteria in surface dust are studied; however, the exact age of surface dust is unknown, and some of it may not have been airborne. Alternatively, Electrostatic Dust Collector (EDC) (ZEEMAN, Alphen, Holland) sampling airborne dust by sedimentation on a cloth has been used in homes, offices and social rooms at workplaces [15–19].

Air is an important transmission route for bacteria and therefore it is important to obtain knowledge about which bacteria are present in the indoor air and which factors affect this presence. In one review paper it was suggested that outdoor bacteria from plants may enter the building through ventilation systems, doors, windows, attached to people, pets and other objects, and as a result, affects the indoor concentration level – but also that information on these factors is still not well understood [20].

Potential health risks of bacteria are for many species evaluated at species level, and matrix-assisted laser desorption time-of-flight mass spectrometry (MALDI-TOF MS) is revealed as a reliable and useful method for identification of bacteria from the indoor environment [1,18]. Using MALDI-TOF MS for identification, it has been shown that the concentration of indoor *Staphylococcus* is associated with the indoor air change rate (ACR) and area per occupant indicating that it might be possible to affect the presence of *Staphylococcus* in indoor air [18]. In contrast, relative humidity (RH) of indoor air was not significantly associated with the concentration of viable bacteria in general [12] and with the commonly present genera *Staphylococcus*, *Bacillus*, *Kocuria* and *Micrococcus* [18].

The aim of this study is to obtain knowledge about which bacterial species (non-*Staphylococcus* species) are present in indoor air in Danish homes, and whether the factors: season, ACR, RH and occupants per area affect the concentration of the most abundant bacterial species in living rooms. To obtain knowledge about whether the concentration of bacteria is related to room type we also study room-to-room variation.

## Methodology

### Study design

All homes were located in the Greater Copenhagen area. Sampling has been performed six or seven times evenly distributed throughout one year in five homes (called 1, 2, 3, 4, 5 with 39, 31, 44, 85 and 66 m^2^/occupant, respectively) in living rooms, bedrooms and bathrooms, and also in the kitchens in three homes, and also in the basements in two homes using samplers called GSPs (Study A, 127 samples in total). Homes 1 and 2 had pets; homes 1–4 had natural ventilation while home 5 had mechanical ventilation; home 4 had previously had moisture problems. In the same five homes and during the same periods, sampling was also performed in living rooms using EDCs (Study B, 20 samples). In study C, EDC samples were taken in another 24 homes in living rooms; these samples were taken in winter (n = 18) or spring (n = 6), with one sample per home. In study D, samples were taken in another 28 homes – also in living rooms using EDC. These samples were taken in the autumn (three samples), winter (20 samples) and spring (five samples).

Dust samples and data on indoor ACR, RH and temperature (temp) were obtained from other studies and they were all measured by members of the research groups [12,18,21,22]. ACR was measured continuously in the five homes (studies A and B) over a 2- to 4-day period following the sampling using GSP (Study A) using the constant concentration methods with a target level of 4 ppm of Freon. The concentration of tracer gas was monitored using an Innova Multi-Gas Monitor Type 1302 and an Innova Multipoint Sampler and Doser 1303 (Lumasense Technologies, Santa Clara, CA, USA). The concentration of tracer gas was separately controlled in the different rooms of each home; for further details see references: [12,22].

In the 24 homes (Study C) ACR was measured using the perfluorocarbon tracer-gas method [23]. In studies A, B and C, RH and temp were measured using Tinytag Plus Data (Gemini, Chichester, UK). In studies A and B, the loggers were placed close to the GSP samplers and set to measure once every 5 min for 15 min between 10:00 and 11:00 am on each sampling day, and average temp and RH were used. In the five homes, the average, ACR, RH and temp were 1.1/h (0.053–5.6), 54.4% (38.9–73.7) and 23.2°C (18.3–26.5), respectively. The ACR and temp were affected by season with highest ACR and temp in summer followed by spring (*P*s < 0.0001). The RH was affected by season with highest RH in autumn followed by summer (*P* < 0.0001). In Study C, loggers measured every 10 min throughout the sampling period and average temp and RH were used. For the 24 homes in Study C, the mean area per occupant, ACR, RH and temp were 15.6 m^2^ (5.2–39.2), 0.54/h (0.1521.23), 29.5% (16.7–44.5) and 22°C (20.3–23.8) [23]. For Study D, no physical data were obtained.

### Sampling and extraction

The GSPs were mounted with polycarbonate filters (37 mm, pore size 1.0 μm; GE Water and Process Technologies, CO, USA), and they sampled airborne bacteria for 6 h from morning to afternoon at a flow rate of 3.5 l/min. The EDC has a surface sampling area of 0.0209 m^2^ (19 × 11 cm) and samples passively. The EDCs were placed on an open surface at 1.2–1.5 m above floor level, allowing dust to settle for 14 days (Study C) or 1 month (Study D). Extraction of dust from EDCs was carried out no later than 24 h post-sample retrieval and extraction of dust from GSP filters no later than 2 h post-sampling. Dust from EDC cloths and GSP filters was extracted according to our previous studies [12]. Briefly, for EDC cloths 20.0 ml pyrogen-free water containing 0.85% sodium chloride (NaCl) and 0.05% Tween 80 was added to a 50 ml tube with one EDC cloth inside and the bacteria were extracted by orbital shaking (500 rpm) for 60 min. For the GSPs the filters were extracted in 5.0 ml of the same solution by orbital shaking (500 rpm) for 15 min, at room temperature. The suspension was harvested and an amount of 1.0 ml of the dust suspension was mixed with 0.5 ml glycerol and kept at –80°C until they were plated on an agar medium.

### Plating and identification of bacteria

An amount of 300 μl of each dust suspension from studies A and B was plated on nutrient agar (NA) plates and incubated at 25°C. From Study C, an amount of 200 μl was plated on NA. All bacterial colonies were counted after 1 week of incubation. In Study A, samples taken at the same time as the samples for Study B were used for identification of bacteria (20 samples). Also from Study A, samples from all rooms from a summer sampling round were used for identification of bacteria (18 samples). From Study B (20 samples), Study C (24 samples) and Study D (28 samples), bacteria in all samples were identified.

Bacterial isolates were identified by MALDI-TOF MS by using a Microflex LT mass spectrometer (Bruker Daltonics, Bremen, Germany). A bacterial test standard (Bruker Daltonics) was used to calibrate the instrument, following the guidelines of the manufacturer. Spectra were analysed using Bruker Biotyper 3.1 software and library. Bacterial isolates were prepared using the extended direct transfer method [24].

### Data treatment

Bacterial concentrations were calculated as time-weighted average (TWA). In the room-to-room study bacterial concentrations are presented as geometric mean (GM) values with confidence limits. The species from EDCs are presented as colony forming units (CFU) of the species/m^2^ per day and GSP data as CFU of the specific species or genera/m^3^ air. Room-to-room variation in bacterial concentrations, RH and temp within rooms was analysed using GLIMMIX with random effects of season, sampling day and home, and using general linear models (GLM). The association between bacterial concentrations (studies A and B), ACR, RH, temp and season were calculated using GLIMMIX with the random effect of the home. Microbial diversity analyses were performed in Rstudio version 3.5.3 using the R CRAN package vegan. Comparisons of microbial diversity between seasons were performed using the analysis of similarity (ANOSIM) function according to Jaccard index values (the index is a measure of the similarity between different sets of data) [25]; a redundancy analysis (RDA) plot using presence–absence were used to visualise community differences between the seasons. The association between bacterial concentrations (Study C), ACR (in the home), area per occupant, RH and temp were also calculated using GLIMMIX. These analyses were done in SAS version 9.4. The most dominating species in terms of bacterial concentration are presented as relative concentrations. Only data on species other than *Staphylococcus* are part of this study.

## Results

### Room-to-room variation

The GM concentrations of bacteria were 519 CFU/m^3^ [253, 1063] across rooms and homes. A room-to-room variation within the five homes was found if data were analysed unaffected by home (*P* = 0.0089) with lowest bacterial concentrations in the cellars (Table 1), and when analysed with random effects of season and home (*P* < 0.0001). In home 1, a high bacterial concentration was found in the bathroom. The temp and RH were different in the different room types. Thus, the temp was lower in the basements and bedrooms than in the other rooms while the RH was high in the bathrooms and basements (Table 1).

**Table 1.** Temperature, RH and ACR, and concentrations of bacteria (GM, CFU/m^3^) in different rooms in homes 1–5

Rooms	n^1)^	Temp. °C		RH %		ACR/h		Across homes CFU bacteria/m^3^		Within homes CFU bacteria/m^3^				
		GM	Range	GM	Range	GM	Range	GM	Cl^2)^	1	2	3	4	5
Bathroom	34	21.2^a,3)^	(16.1–27.2)	60^a^	(35.5–84.0)	Nm^5)^		761^a^	[417, 1386]	1088^a^	695^a^	389^a^	2479^a^	414^ab^
Basement	13	18.0^c^	(13.9–24.6)	63^a^	(40.0–81.3)	0.088^b,6)^	(0.017–0.32)	144^b^	[62, 336]	-	98^b^	-	225^b^	-
Bedroom	34	20.1^b^	(15.1–27.3)	55^b^	(32.1–76.1)	0.586^a^	(0.022–6.1)	746^a^	[366, 1520]	609^b^	433^ab^	700^a^	2047^a^	706^a^
Kitchen	13	22.5^a^	(19.8–26.3)	54^b^	(40.5–68.4)	0.328^ab^	(0.043–2.0)	279^a^	[118, 662]	478^b^	-	176^a^	-	-
Living room	33	21.3^a^	(17.1–28.6)	57^b^	(45.7–77.8)	0.307^ab^	(0.0004–5.6)	509^a^	[278, 934]	309^b^	1015^a^	273^a^	1469^a^	295^b^
*P*-values^4)^		<0.0001		0.0003		0.23		<0.0001		0.078	0.0038	0.23	0.019	0.087

^1)^n = numbers of samples. ^2)^Cl = confidence limit. ^3)^Numbers in the same column followed by the same letter are not significantly different. ^4)^*P*-values for comparisons of physical factors or bacterial concentrations between room types. ^5)^Not measured. ^6)^n = 6.

In one summer sampling round, the bacteria from the different rooms were identified. The three bacterial species found in the highest concentrations in all rooms were the same within the same home; in all homes, *M. luteus* was among these three species. Other dominating species are mentioned in brackets (home 1: *Sphingomonas aerolata* and *Lysinibacillus*; home 2: *Bacillus licheniformis* and *Arthrobacter sulfonivorans*; home 3: *M. osloensis* and *P. yeei*; home 4: *K. palustris* and *K. rhizophila*; home 5: *M. osloensis* and *P. yeei.*

### Bacterial species in living rooms

**Figure 1:**
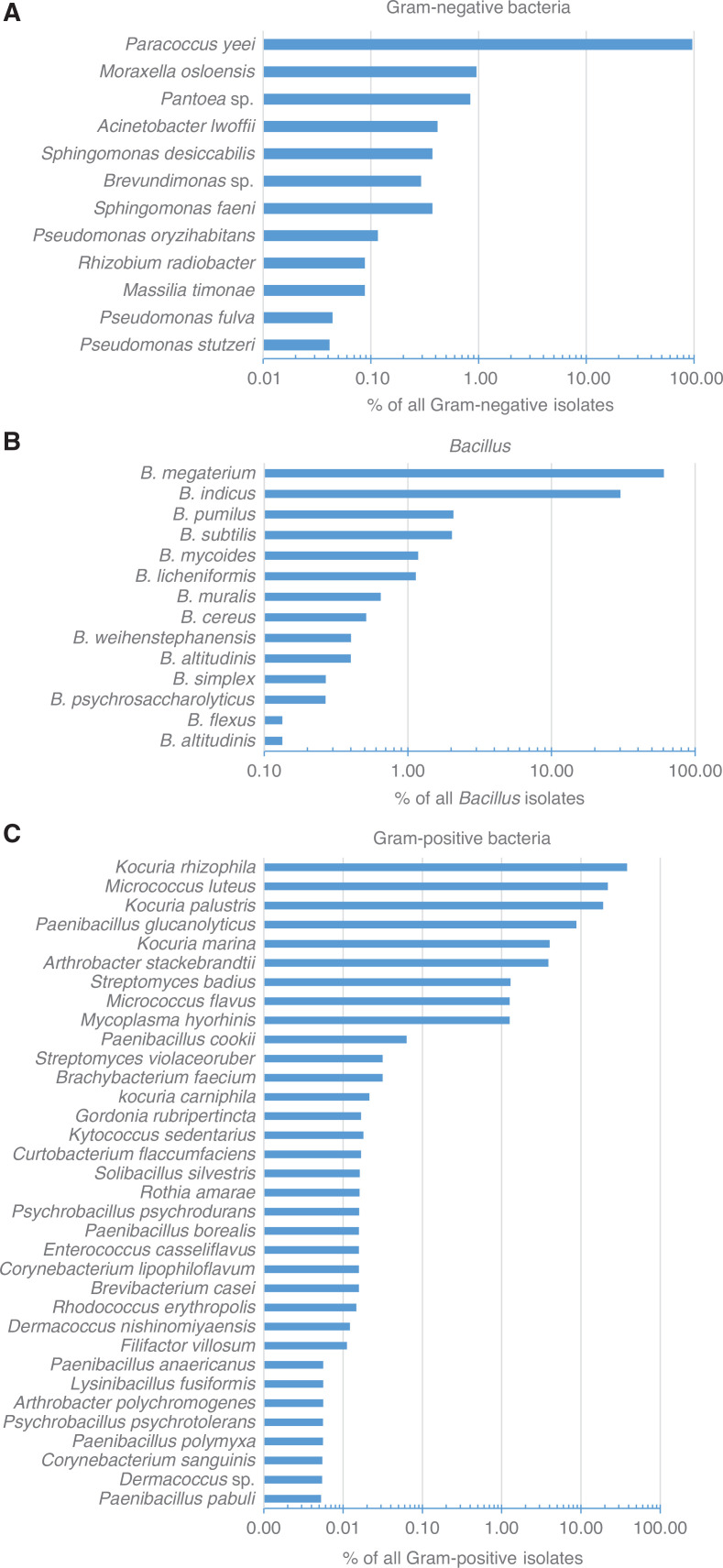
(A—C) Gram-negative bacteria (A), the 14 *Bacillus* species (B) and Gram-positive bacteria other than *Bacillus* and *Staphylococcus* (C) found in the living rooms of the five homes with one sample from each season (Study A); all presented as % of total isolates within the category. (Source: Authors, 2023.)

Bacteria were identified in one sample per season in the living rooms of the five homes (Study A). Some species were observed frequently, but in low concentrations, for example, *M. osloensis*, while other species, for example, *B. megaterium* and *Paenibacillus glucanolyticus* were seldom observed but when observed they were present in high concentrations. The Gram-negative bacterium *P. yeei* was observed repeatedly, and it constituted a large part of the airborne Gram-negative bacteria (Fig. 1A). Fourteen different *Bacillus* species were found (Fig. 1B). Of Gram-positive bacteria (other than *Bacillus* species) the species *K. rhizophila* was found in many samples and in high concentrations (Fig. 1C).

**Figure 2:**
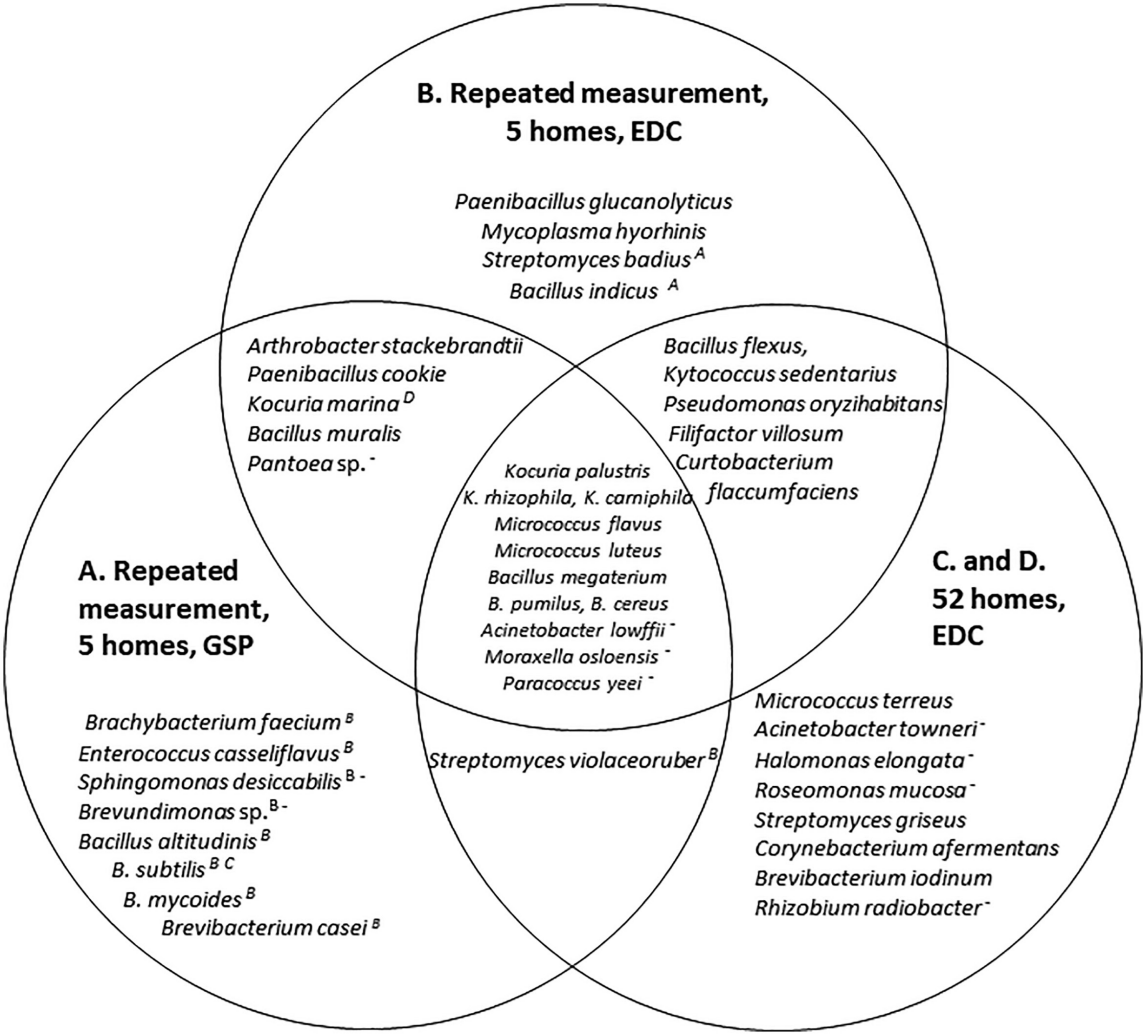
The 25 dominating bacterial species in samples from the living room in five homes with repeated measurements using GSPs (A) for sampling once per season using EDCs (B), and once in the living room of 24 (C) and 28 (D) homes using EDCs. The A, B, C and Ds following some bacterial names indicate that the bacterium has also been found in studies A, B, C or D – but not among the 25 dominating species, and the symbol ‘–’ indicates that the bacterium is Gram-negative. (Source: Authors, 2023.)

**Figure 3:**
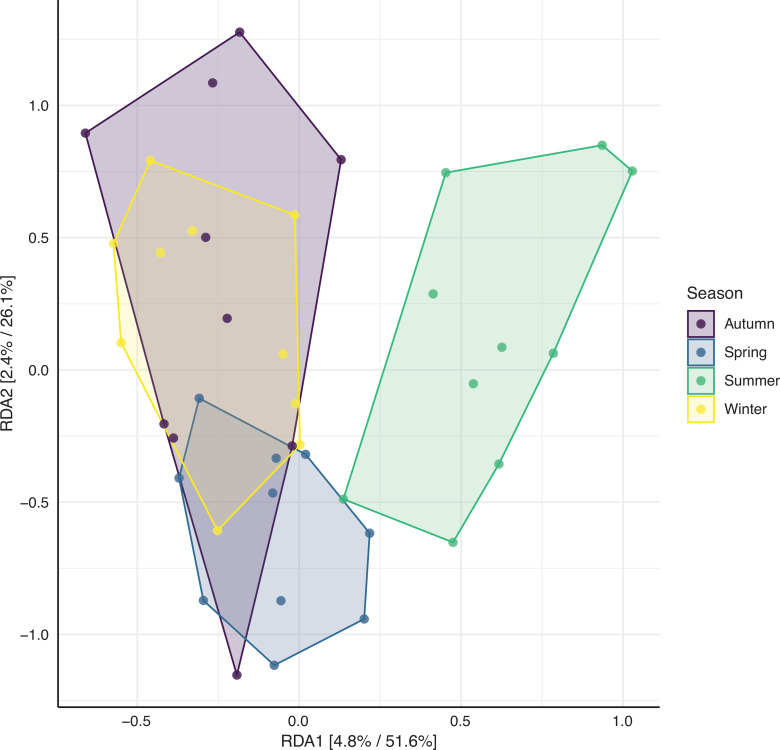
RDA (redundancy analysis) plotting of airborne bacteria in homes constrained by the season; circles represent an individual sample. Percentages on the axes refer to the relative contribution (eigenvalue) of each axis to the total inertia in the data and the relative contribution of the particular axis to the total constrained space. Samples are coloured by season, and a polygon is drawn around samples representing the same season. ANOSIM: R^2^ = 0.08, *P* = 0.009. (Source: Authors, 2023.)

To see whether there is an overlap in bacterial species as sampled using GSPs during 1 day each season (Study A) versus using EDC samplers for long-term sampling (Study B) the bacterial species found in the highest concentrations in the five homes are presented in a Venn diagram and an overlap in species is found (Fig. 2). In addition, irrespective of whether samples were taken using an EDC or GSP sampler, the microbial diversity varied between seasons (Fig. 3).

To get an impression of bacterial species in living rooms of Danish city homes in general, 52 samples taken using EDCs were screened for bacterial species (studies C and D), and the 25 species found in highest concentrations are presented in the Venn diagram. Eleven species were found in high concentrations in the living rooms of the five homes unaffected by the sampling method and in the 52 homes (Fig. 2). A list of species found in more than one home can be found in Table A1.

### Concentrations of selected bacterial species as affected by indoor physical factors

For some of the species we have found most frequently and for Gram-negative species considered together, we have studied factors which may affect the measured concentration. Associations with *P*-values < 0.1 are considered significant. In Study A, the concentration of Gram-negative bacteria as measured using the GSP samplers was associated with the season (*P* = 0.0016) with lower concentrations in summer than in winter. The concentration of *P. yeei* tended to be associated negatively with increasing temperature and was associated with the season with the highest concentrations in spring. *Micrococcus flavus* was negatively associated with ACR and *B. pumilus* positively with RH. *Kocuria rhizophila* was positively associated with RH and negatively with temperature and ACR (Table 2).

**Table 2.** Associations between concentrations of airborne bacteria as measured using GSP samplers repeatedly in the living room of five homes, and ACR, RH, temp and season (Study A)

Fixed factor	*Paracoccus yeei*		*Bacillus pumilus*		*Kocuria palustris*		*Kocuria rhizophila*		*Micrococcus luteus*		*Micrococcus flavus*	
	Estimate	*P*-value^1)^	Estimate	*P*-value	Estimate	*P*-value	Estimate	*P*-value	Estimate	*P*-value	Estimate	*P*-value
Each factor studied separately												

ACR (room)	−0.41	0.39	−0.24	0.40	−0.14	0.52	**−0.37**	**0.091**	−0.26	0.12	**−0.48**	**0.059**
RH	2.40	0.52	**6.78**	**0.0002**	**5.21**	**0.096**	**7.44**	**0.022**	−0.17	0.94	−0.26	0.95
Temperature	−5.56	0.15	−0.046	0.31	**−0.12**	**0.048**	**−0.14**	**0.051**	−0.0075	0.11	−0.017	0.84
Season^3)^	**-**	0.081	**-**	**0.0045**	-	**0.019**	**-**	**0.069**	**-**	0.077	-	0.63
Spring	**1.46**	**0.042**	0.060	0.85	−0.27	0.59	−0.40	0.52	0.49	0.26	0.10	0.90
Summer	−0.25	0.70	0.060	0.85	−0.50	0.34	−0.71	0.26	−0.58	0.18	−0.75	0.37
Autumn	0.18	0.78	**1.19**	**0.0019**	**1.21**	**0.031**	0.98	0.13	0.42	0.34	0.22	0.79
Winter	reference		reference		reference		reference		reference		reference	

Stepwise regression^2)^												

ACR (room)	**-**	**-**	**-**	**-**	**-**	**-**	**−0.51**	**0.0059**	**-**	**-**	**−0.48**	**0.059**
RH	**8.85**	**0.064**	**6.78**	**0.0002**	**-**	-	**8.70**	**0.0025**	**-**	**-**	**-**	**-**
Temperature	**-**	**-**	**-**	-	**-**	-	-	-	**-**	**-**	**-**	**-**
Season	**-**	**0.036**	**-**	-	**-**	**0.019**	-	-	**-**	**-**	**-**	**-**
Spring	**1.54**	**0.034**	**-**	-	**-**	-	-	-	**-**	**-**	**-**	**-**
Summer	**-**	**-**	**-**	-	**-**	-	-	-	**-**	**-**	**-**	**-**
Autumn	**-**	**-**	**-**	-	**1.21**	**0.031**	-	-	**-**	**-**	**-**	**-**
Winter	reference		reference		reference		reference		reference		reference	

^1)^*P*-values below 0.1 are in bold; concentrations are compared using the GLIMMIX procedure with Poisson distributed data; estimates (β-coefficients) are presented. ^2)^Statistically significant factors in the stepwise regression with backward regression. ^3)^Relative to winter.

In Study B, *K. rhizophila* was associated negatively with temperature (*β* = −10.0, *P* = 0.031); other associations were not significant. In the cross-sectional study (Study C), associations were found between area per occupant and *K. palustris* concentration (*β* = −1.6, *P* = 0.091), and temperature and *M. flavus* concentration (*β* = −21, *P* = 0.079).

## Discussion

In this study, we show that cultivable *K. palustris*, *K. rhizophila*, *M. luteus*, *M. flavus*, *B. pumilus*, *K. megaterium*, *K. carniphila*, *A. lowffii*, *M. osloensis* and *P. yeei* are common in indoor air in homes in Greater Copenhagen, in addition to the previously found *Staphylococcus* species [18]. These results were stable and unaffected by the sampling method. Thus, people seem to be exposed to these bacteria via inhalation on daily basis.

Some of the found species are classified as risk class 2 pathogens, which means that they can cause human disease but are unlikely to spread to the community, and there is usually an effective prophylaxis or treatment available [26]; for example, *P. yeei*, which has caused, for example, keratitis and conjunctivitis [27,28] and *B. cereus*, which is a food-poisoning agent [26]. Examples of other risk class 2 pathogens found in the homes are: *Aerococcus viridans*, *Acinetobacter lwoffii*, *Bacillus mycoides*, *Brevibacterium casei*, *Enterococcus casseliflavus* and several *Kocuria* species. Some of the risk class 2 pathogens are normal skin-related bacteria such as *B. casei* and are expected to derive from the occupants. No risk class 3 pathogens were found. Some species are described as opportunistic pathogens, for example, *M. osloensis* (e.g., in the airways of an elderly patient [29]) and *Rhizobium radiobacter* (e.g., pneumonia in a cancer patient [30]). An underlying mechanism explaining many health effects of exposure to airborne particles is the ability to induce the formation of reactive oxygen species (ROS) within the airways. There is limited knowledge on the ability of different species to induce ROS production (without infection), but few studies indicate differences at the species level [31,32].

Many of the species found have previously been found in very different environments. The bacteria found in high concentrations, *P. yeei*, *B. megatarium* and *M. luteus*, have previously been found on indoor surfaces [33,34], but also as airborne bacteria in totally different environments such as wastewater treatment plants [35], pigeon coops [36] and on workers’ clothes [37]. We have found many different *Bacillus* species, and a high species richness of *Bacillus* has also been found in air samples from different occupational settings [36,38] as well as on indoor surfaces [33]. When considering the bacteria at the genus level, the genera *Kocuria*, *Micrococcus*, *Bacillus* and *Paenibacillus* were among the dominating ones, and these genera were also dominating in indoor air in Hong Kong and China (reviewed in [20]).

This study found a within-home variation (room-to-room) for concentrations of airborne bacteria. Thus, airborne bacterial concentrations were not uniform throughout the homes during the time of sampling, which is in agreement with a study in homes in the UK [39]. In spite of this, no significant variation was observed for general room type (e.g., bathroom vs. living room) except for a lower concentration in basements. Furthermore, the same bacterial species were found in the highest concentrations in all rooms within the same home. In a study from the USA, bacterial concentrations in cellars were lower than in bathrooms, while cellars, kitchens and bedrooms did not differ significantly [14]. In this study, there was a tendency towards the highest bacterial concentrations and humidity in bathrooms, while bedrooms also had high bacterial concentrations but low temperatures. This may indicate a larger contribution of skin-related bacteria such as *Corynebacterium xerosis* and *Dermacoccus* sp. to the airborne bacteria in these rooms. However, it was only a tendency, and the lack of a general effect of room type for bacterial concentration is in accordance with what was found in Chinese homes [40]. This indicates, on the one hand, that the variation in exposure found between rooms may not be attributable to what the rooms are used for in general, or that it is also affected by other factors such as, for example, ACR. In this study, the repeated sampling using GSP samplers in the five homes was done in the daytime while for the most part the occupants were not at home. Previous studies have shown that bacterial concentrations in indoor air are higher in the presence of occupants [21].

The bacterial diversity in the living rooms differed between seasons with summer especially having another bacterial diversity, this might be caused by the high ACR in the summer. For some bacterial species, associations between concentrations in living rooms and seasons were found. Thus, *P. yeei* was found in the highest concentrations in the spring. The habitat of *P. yeei* seems not to be well characterised, and in research papers it is mainly described concerning infections. Therefore, we do not know the source of exposure to this bacterium. *Paracoccus yeei* is a Gram-negative bacterium, and for Gram-negative bacteria in general lower concentrations were found in summer than in winter. This may be related to the impact of ultraviolet (UV) light on bacterial survival.

The two species *B. pumilus* and *K. palustris* were found in the highest concentrations in the autumn. These bacteria have previously been found in soil. At the genus level, we have previously observed that *Kocuria* is present in the lowest concentrations in summer [18]. *Kocuria palustris*, *K. rhizophila* and *M. flavus* were associated negatively with indoor temperature. For the two *Kocuria* species, this is in accordance with what has previously been found for the genus and in contrast to what is found for the genus *Staphylococcus* in Danish homes [18] and bacteria in general in Greek homes [41].

Human skin is shed into the indoor air [42], and therefore it could be expected that the concentration of skin-related bacterial species is negatively associated with ACR. The two species *K. rhizophila* and *M. flavus* were negatively associated with ACR and thus seem not to enter by open windows or ventilation systems. These species are not described as skin-related bacteria, but *K. rhizophila* has been found on the skin [43]. Another transmission route to the home environment may be clothing and in particular work clothing from environments with high exposure to bacteria. Thus, a recent study has shown that bacteria accumulate on work clothing in high amounts during a workday and that bacteria may be released from the clothes to the home air; in fact, cultivable *K. rhizophila* has been found on work clothes together with more than 200 different cultivable bacterial species [37,44]. In Study C, the *K. palustris* concentration was negatively associated with area per occupant. Therefore, it may also have human or human activity as a source. The habitats of this species are not well described, but it has been isolated from very different environments including human skin [45], workers’ hands [24], human noses [46] and marine algae [47]. In a study about bacterial genera in outdoor air, *Bacillus* and *Acinetobacter* but not *Kocuria* were among the most frequently found genera [48].

*Micrococcus luteus* was very common in indoor air in this study, and it is described as a skin-related bacterium. Despite that, it was not significantly associated with ACR or area per occupant. Furthermore, the species did not show seasonality. The lack of association between the studied factors and concentrations of *M. luteus* might be because this species has several sources as it is found in soil, dust [49], airways and on human skin [50]. It has also been found in the air in schools [49] in the air and on hand palms in occupational settings [24].

## Conclusion

Across homes and room types within homes, occupants are potentially exposed to some of the same cultivable bacterial species typically including: *K. palustris*, *K. rhizophila*, *M. luteus*, *M. flavus*, *B. pumilus*, *B. megaterium*, *K. carniphila*, *A. lowffii*, *M. osloensis* and *P. yeei*. Seasonality in bacterial diversity was found, and concentrations of *P. yeei* were significantly associated with the season. Bacterial concentrations were not uniform throughout the homes investigated, but no significant variation was observed for the general room type except for the lower concentration in the basements. The concentrations of *P. yeei*, *K. rhizophila* and *B. pumilus* were positively associated with RH, and concentrations of *K. rhizophila* were associated negatively with temp while *K. rhizophila* and *M. flavus* were negatively associated with ACR, and *K. palustris* negatively with area per occupant. Thus decreasing the RH, and increasing the ACR and area per occupant might be a strategy to reduce the exposure to some airborne bacterial species.

## Data Availability

The datasets generated during and/or analysed during the current study are available from the corresponding author on reasonable request.

## Appendix

### Appendix A

**Table A1.** Bacterial species found in more than one of 57 homes

*Acinetobacter lwoffii*	*Brevibacterium casei*	*Micrococcus luteus*
*Acinetobacter schindleri*	*Brevibacterium iodinum*	*Micrococcus terreus*
*Acinetobacter towneri*	*Brevundimonas* sp.	*Moraxella osloensis*
*Aerococcus viridans*	*Celluslosimicrobium cellulans*	*Mycoplasma hyorhinis*
*Arthrobacter oxydans*	*Corynebacterium afermentans*	*Paenibacillus amylolyticus*
*Arthrobacter polychromogenes*	*Corynebacterium lipophiloflavum*	*Paenibacillus anaericanus*
*Arthrobacter stackebrandtii*	*Corynebacterium sanguinis*	*Paenibacillus borealis*
*Arthrobacter sulfonivorans*	*Curtobacterium flaccumfaciens*	*Paenibacillus cookii*
*Bacillus altitudinis*	*Dermacoccus nishinomiyaensis*	*Paenibacillus glucanolyticus*
*Bacillus amyloliquefaciens*	*Dermacoccus* sp.	*Paenibacillus pabuli*
*Bacillus arsenicus*	*Dietzia cinnamea*	*Paenibacillus polymyxa*
*Bacillus badius*	*Dietzia papillomatosis*	*Pantoea* sp.
*Bacillus cereus*	*Enterococcus casseliflavus*	*Paracoccus* sp.
*Bacillus clausii*	*Filifactor villosum*	*Paracoccus yeei*
*Bacillus firmus*	*Gordonia rubripertincta*	*Penicillium brevicompactum*
*Bacillus flexus*	*Halomonas elongata*	*Pseudomonas fulva*
*Bacillus indicus*	*Jeotgalicoccus halotolerans*	*Pseudomonas oryzihabitans*
*Bacillus iodinum*	*Kocuria carniphila*	*Pseudomonas stutzeri*
*Bacillus licheniformis*	*Kocuria marina*	*Psychrobacillus*
*Bacillus megaterium*	*Kocuria palustris*	*Psychrobacillus psychrotolerans*
*Bacillus muralis*	*Kocuria rhizophila*	*Rhizobium radiobacter*
*Bacillus mycoides*	*Kocuria* sp.	*Rhodococcus corynebacterioides*
*Bacillus psychrosaccharolyticus*	*Kocuria varians*	*Rhodococcus erythropolis*
*Bacillus pumilus*	*Kytococcus schroeteri*	*Rhodococcus kroppenstedtii*
*Bacillus simplex*	*Kytococcus sedentarius*	*Roseomonas mucosa*
*Bacillus subtilis*	*Lactobacillus sakei*	*Rothia amarae*
*Bacillus vallismortis*	*Lysinibacillus fusiformis*	*Solibacillus silvestris*
*Bacillus weihenstephanensis*	*Lysinibacillus sphaericus*	*Sphingomonas desiccabilis*
*Bacillus licheniformis*	*Macrococcus caseolyticus*	*Sphingomonas faeni*
*Bacillus subtilis*	*Massilia timonae*	*Sphingomonas* sp.
*Bacillus psychrosaccharolyticus*	*Microbacterium oxydans*	*Sporosarcina psychrophila*
*Brachybacterium faecium*	*Microbacterium paraoxydans*	*Streptomyces badius*
*Brevibacillus agri*	*Micrococcus flavus*	*Streptomyces griseus*
		*Streptomyces violaceoruber*

## References

[1] Mkrtchyan HV, Russell CA, Wang N, Cutler RR (2013). Could public restrooms be an environment for bacterial resistomes?. PLoS ONE.

[2] Perkins SD, Angenent LT (2010). Potential pathogenic bacteria in metalworking fluids and aerosols from a machining facility. FEMS Microbiol Ecol.

[3] Atosuo J, Karhuvaara O, Suominen E, Vilén L, Nuutila J, Putus T (2021). Indoor-related microbe damage induces complement system activation in building users. Innate Immun.

[4] Ege MJ, Mayer M, Normand A-C, Genuneit J, Cookson WO, Braun-Fahrländer C (2011). Exposure to environmental microorganisms and childhood asthma. N Engl J Med.

[5] Brooks C, Pearce N, Douwes J (2013). The hygiene hypothesis in allergy and asthma: an update. Curr Opin Allergy Clin Immunol.

[6] Overton E (1988). The Journal of Infection Control Nursing. Bed-making and bacteria. Nurs Times.

[7] Balasubramanian R, Nainar P, Rajasekar A (2012). Airborne bacteria, fungi, and endotoxin levels in residential microenvironments: a case study. Aerobiologia.

[8] Gorny RL, Dutkiewicz J (2002). Bacterial and fungal aerosols in indoor environment in Central and Eastern European countries. Ann Agric Environ Med.

[9] Bartlett KH, Kennedy SM, Brauer M, Van Netten C, Dill B (2004). Evaluation and a predictive model of airborne fungal concentrations in school classrooms. Ann Occup Hyg.

[10] Scheff PA, Paulius VK, Curtis L, Conroy LM (2000). Indoor air quality in a middle school, Part II: development of emission factors for particulate matter and bioaerosols. Appl Occup Environ Hyg.

[11] Kalogerakis N, Paschali D, Lekaditis V, Pantidou A, Eleftheriadis K, Lazaridis M (2005). Indoor air quality-bioaerosol measurements in domestic and office premises. J Aerosol Sci.

[12] Frankel M, Beko G, Timm M, Gustavsen S, Hansen EW, Madsen AM (2012). Seasonal variation of indoor microbial exposures and their relations to temperature, relative humidity and air exchange rates. Appl Environ Microbiol.

[13] Adhikari A, Lewis JS, Reponen T, Degrasse EC, Grimsley LF, Chew GL (2010). Exposure matrices of endotoxin, (1→3)-β-d-glucan, fungi, and dust mite allergens in flood-affected homes of New Orleans. Sci Total Environ.

[14] Moschandreas D, Pagilla KR, Storino LV (2003). Time and space uniformity of indoor bacteria concentrations in Chicago area residences. Aerosol Sci Technol.

[15] Rocchi S, Reboux G, Frossard V, Scherer E, Valot B, Laboissière A (2015). Microbiological characterization of 3193 French dwellings of Elfe cohort children. Sci Total Environ.

[16] Frankel M, Timm M, Hansen EW, Madsen AM (2012). Comparison of sampling methods for assessment of indoor microbial exposure. Indoor Air.

[17] Noss I, Wouters IM, Visser M, Heederik DJ, Thorne PS, Brunekreef B (2008). Evaluation of a low-cost electrostatic dust fall collector for indoor air endotoxin exposure assessment. Appl Environ Microbiol.

[18] Madsen AM, Moslehi-Jenabian S, Islam MZ, Frankel M, Spilak M, Frederiksen MW (2018). Concentrations of *Staphylococcus* species in indoor air as associated with other bacteria, season, relative humidity, air change rate, and *S. aureus*-positive occupants. Environ Res.

[19] Liebers V, van KV, Bunger J, Düser M, Stubel H, Brüning T (2012). Assessment of airborne exposure to endotoxin and pyrogenic active dust using electrostatic dustfall collectors (EDCs). J Toxicol Environ Health A.

[20] Guo K, Qian H, Zhao D, Ye J, Zhang Y, Kan H (2020). Indoor exposure levels of bacteria and fungi in residences, schools, and offices in China: a systematic review. Indoor Air.

[21] Madsen AM, Matthiesen CB, Frederiksen MW, Frederiksen M, Frankel M, Spilak M (2012). Sampling, extraction and measurement of bacteria, endotoxin, fungi and inflammatory potential of settling indoor dust. J Environ Monit.

[22] Bekö G, Gustavsen S, Frederiksen M, Bergsøe NC, Kolarik B, Gunnarsen L (2016). Diurnal and seasonal variation in air exchange rates and interzonal airflows measured by active and passive tracer gas in homes. Build Environ.

[23] Spilak MP, Madsen AM, Knudsen SM, Kolarik B, Hansen EW, Frederiksen M (2015). Impact of dwelling characteristics on concentrations of bacteria, fungi, endotoxin and total inflammatory potential in settled dust. Build Environ.

[24] Madsen AM, Frederiksen MW, Jacobsen MH, Tendal K (2020). Towards a risk evaluation of workers’ exposure to handborne and airborne microbial species as exemplified with waste collection workers. Environ Res.

[25] Oksanen F, Blanchet M, Friendly R, Kindt P, Legendre D, McGlinn P (2018). Solymos, Vegan: Community Ecology Package. R package version 2.5–3.

[26] Unfallversicherung IfAdDG (2017). Gestis – Internationale Grenzwerte für chemische Substanzen.

[27] Courjaret J-C, Drancourt M, Hoffart L (2014). Paracoccus yeei keratitis in a contact lens wearer. Eye Contact Lens.

[28] Ufford I, Caines J, Haigh H, Wilson C (2015). *Paracoccus yeei* species causing bacterical conjuctivitis with cellulitis. Med Res Arch.

[29] Gargiulo C, Van Hung P, Hai NT, Nguyen KCD, Davey KF, Abe K (2015). A case report of an elderly patient with respiratory failure caused by *Moraxella osloensis* infection. Anaesthesia.

[30] Lai C-C, Teng L-J, Hsueh P-R, Yuan A, Tsai KC, Tang JL (2004). Clinical and microbiological characteristics of *Rhizobium radiobacter* infections. Clin Infect Dis.

[31] Samake A, Uzu G, Martins J, Calas A, Vince E, Parat S (2017). The unexpected role of bioaerosols in the Oxidative Potential of PM. Sci Rep.

[32] Hirvonen MR, Huttunen K, Roponen M (2005). Bacterial strains from moldy buildings are highly potent inducers of inflammatory and cytotoxic effects. Indoor Air.

[33] Madsen AM, Phan HU, Laursen M, White JK, Uhrbrand K (2020). Evaluation of methods for sampling of *Staphylococcus aureus* and other *Staphylococcus* species from indoor surfaces. Ann Work Expo Health.

[34] Bae IK, Kang DH, Kim SM, Bae YM (2017). Distribution and identification of gram-negative bacteria in indoor environment of houses. J Korean Soc Oral Health Sci.

[35] Lu R, Frederiksen MW, Uhrbrand K, Li Y, Østergaard C (2020). Wastewater treatment plant workers’ exposure and methods for risk evaluation of their exposure. J Ecotoxicol Environ Saf.

[36] Madsen AM, White JK, Nielsen JL, Keskin ME, Tendal K, Frederiksen MW (2022). A cross sectional study on airborne inhalable microorganisms, endotoxin, and particles in pigeon coops – risk assessment of exposure. Environ Res.

[37] Madsen AM, Rasmussen PU, Frederiksen MW (2022). Accumulation of microorganisms on work clothes of workers collecting different types of waste – a feasibility study. Waste Manag.

[38] Madsen AM, White JK, Markouch A, Kadhim S, de Jonge N, Thilsing T (2020). A cohort study of cucumber greenhouse workers’ exposure to microorganisms as measured using NGS and MALDI-TOF MS and biomarkers of systemic inflammation. Environ Res.

[39] Nasir ZA, Colbeck I (2012). Winter time concentrations and size distribution of bioaerosols in different residential settings in the UK. Water Air Soil Pollution.

[40] Ye J, Qian H, Zhang J, Sun F, Zhuge Y, Zheng X (2021). Combining culturing and 16S rDNA sequencing to reveal seasonal and room variations of household airborne bacteria and correlative environmental factors in Nanjing, Southeast China. Indoor air.

[41] Stamatelopoulou A, Pyrri I, Asimakopoulos D, Maggos T (2020). Indoor air quality and dustborne biocontaminants in bedrooms of toddlers in Athens, Greece. Build Environ Int.

[42] Noble WC, Habbema JD, van Furth R, Smith I, de Raay C (1976). Quantitative studies on the dispersal of skin bacteria into the air. J Med Microbiol.

[43] Hillion M, Mijouin L, Jaouen T, Barreau M, Meunier P, Lefeuvre L (2013). Comparative study of normal and sensitive skin aerobic bacterial populations. Microbiologyopen.

[44] Møller SA, Rasmussen PU, Frederiksen MW, Madsen AM (2022). Work clothes as a vector for microorganisms: accumulation, transport, and resuspension of microorganisms as demonstrated for waste collection workers. Environ Int.

[45] Al Bayatee MA, Alsammak EG (2018). Phenetic and phylogenetic analysis of *Kocuria palustris* and *Kocuria rhizophila* strains isolated from healthy and thalassemia persons. Sci J Med Res.

[46] Kaspar U, Kriegeskorte A, Schubert T, Peters G, Rudack C, Pieper DH (2016). The culturome of the human nose habitats reveals individual bacterial fingerprint patterns. Environ Microbiol.

[47] Leiva S, Alvarado P, Huang Y, Wang J, Garrido I (2015). Diversity of pigmented Gram-positive bacteria associated with marine macroalgae from Antarctica. FEMS Microbiol Lett.

[48] Fykse EM, Tjärnhage T, Humppi T, Eggen VS, Ingebretsen A, Skogan G (2015). Identification of airborne bacteria by 16S rDNA sequencing, MALDI-TOF MS and the MIDI microbial identification system. Aerobiologia (Bologna).

[49] Fox K, Fox A, Elssner T, Feigley C, Salzberg D (2010). MALDI-TOF mass spectrometry speciation of staphylococci and their discrimination from micrococci isolated from indoor air of schoolrooms. J Environ Monit.

[50] Kloos WE, Musselwhite MS (1975). Distribution and persistence of *Staphylococcus* and *Micrococcus* species and other aerobic bacteria on human skin. Appl Microbiol.

